# Social Media Engagement Among Oculoplastic Surgeons in India: Patterns and Perceptions

**DOI:** 10.7759/cureus.80710

**Published:** 2025-03-17

**Authors:** Akshay Gopinathan Nair

**Affiliations:** 1 Ophthalmic Plastic Surgery and Ocular Oncology, Advanced Eye Hospital and Institute, Mumbai, IND

**Keywords:** facebook, healthcare social media, instagram, instagram followers panel, ophthalmology, plastic surgery, social media

## Abstract

Aim: Social media engagement in the form of sharing before and after photographs following surgery and posting surgical videos and patient testimonials has become a popular form of promotion and marketing for clinicians. A significant proportion of patients undergoing cosmetic surgery are influenced by the results or before/after photos. Different geographical regions, though, have variations both among surgeons posting content and the viewers being influenced by them. The aim of the survey was to assess the patterns of social media use for professional promotion and patient outreach and the self-reported perceived impact of social media among oculoplastic surgeons in India.

Methods: An online survey was sent to members of the Oculoplastics Association of India (OPAI) in early 2023.

Results: This was an anonymized survey and had a response rate of 36% (252/702). Of the 252 responses, 66% were women. In all, 28.6% of the respondents had their own professional website. When asked if they felt social media engagement was an important part of practice building, 73% agreed, 9% disagreed, and 19% were unsure. However, only 34% of the respondents had a social media presence related to their practice/professional account. Of those with a social media presence, the most popular platform was Facebook (77%), followed by Instagram (72%) and YouTube (48%). In all, 52/86 (61%) surgeons reported that social media posts had translated into patient visits. Posts on ptosis and blepharoplasty resulted in maximum engagement and patient visits. Of those who did not have a social media presence, constant pressure to post content regularly and unfamiliarity with the platform were the most common reasons cited. Also, 65% of the respondents under the age of 40 reported having a professional practice-related social media profile as compared to 31% of those above 40 (p<0.0001).

Conclusions: Members of OPAI largely see social media engagement as an important part of practice building, but unfamiliarity with the platform and constant pressure to post content are challenges faced by them. Social media presence appears to benefit some surgeons by way of an increase in clinic visits and improved visibility. Facebook and Instagram remain the most popular platforms favored by the OPAI members. The proportion of oculoplastic surgeons below 40 years of age who are active on social media is significantly higher than those aged over 40 (p<0.0001).

## Introduction

In recent years, the influence of social media has permeated various professional domains, including medicine and, specifically, the subspecialty of ophthalmology. The ability to connect with a broader audience, disseminate knowledge, and engage with peers and potential patients on platforms such as Facebook, Instagram, and YouTube has become increasingly relevant for medical practitioners. As the digital age continues to reshape the dynamics of professional communication and patient outreach, it is imperative to assess the usage patterns, trends, motivations, and challenges faced by these practitioners in navigating the realm of social media.

The utilization of social media platforms in the healthcare sector, and more so in ophthalmology, is not a novel concept. Previously published studies have shown that the majority of ophthalmology content on Instagram, one of the most popular social media platforms, is authored by non-ophthalmologists, with educational content being the least engaging [1]. The Internet remains the most frequently used source of health information by patients. It has been reported that close to 90% of young adults use social media, which has resulted in nearly 2.5 billion monthly Facebook users and over one billion monthly users on Instagram [2]. As a result, many oculoplastic surgeons around the world have incorporated social media promotion and medical content creation as a part of building and expanding their brand and practice. This is because highlighting the results of surgeries, especially aesthetic and cosmetic procedures, can serve as an extremely powerful marketing tool. These social network platforms allow surgeons to showcase their practices and create patient-awareness content that prospective patients may be seeking to know before electing to undergo surgery. Ophthalmology, and more specifically oculoplastics, are highly visual branches, which allow eye-related discussions to be shared on image- and video-based public platforms such as Instagram and YouTube [1]. It is not uncommon to see many oculoplastic and facial plastic surgeons publish preoperative and postoperative results to showcase their surgical results on their popular channels to highlight the different treatment options that they offer [2]. Many studies have tried to assess the impact and chart the practice patterns of usage of social media in surgical specialties. However, to the best of my knowledge, there has been no survey from India that has studied the current trends of social media usage by oculoplastic surgeons. With this background, this study was designed to assess the evolving landscape of social media engagement among oculoplastic surgeons in India. This article was previously presented as a poster at the 2023 Annual Meeting of the Oculoplastics Association of India in Hyderabad, India.

## Materials and methods

A link to an anonymized survey that included questions on social media usage was sent in June 2023 to members of the Oculoplastic Association of India (OPAI) through email communication and WhatsApp over various groups of OPAI. The email/message clearly explained the nature of the survey and its questions and contained a hyperlink to an electronic survey hosted by a third-party website, Google Forms (Microsoft Corp., Redmond, USA). Subsequently, a reminder to take the survey was sent after two weeks. The survey contained 21 questions, most of which were multiple-choice questions. In the context of this study, "professional promotion" refers to the use of social media platforms by oculoplastic surgeons to showcase their expertise, share surgical outcomes, and highlight their practice through before and after photographs, surgical videos, and patient testimonials. This form of promotion aims to increase visibility, attract potential patients, and build a professional reputation. "Patient outreach" refers to the efforts made by surgeons to engage with current and potential patients through social media platforms. This includes sharing educational content, raising awareness about oculoplastic procedures, and providing information that helps patients make informed decisions about their treatment options.

The survey began with a written consent form, which was presented to the respondents who clicked on the hyperlink. This consent form mentioned that participation in the survey was entirely voluntary. All respondents were informed that the collected responses would be confidential. Additionally, no identifiable information such as name, IP address, or email address would be collected from the participants. Only after consenting to this were the respondents allowed to proceed to the questions of the survey. Respondents were asked questions that included demographic information of the respondent regarding years of practice, age, and practice setting. Answering all questions in the survey was mandatory. The survey was anonymized and did not contain any identifying information. Institutional Review Board approval was requested and waived as the study did not involve intervention in human subjects. Association between categorical variables was assessed using Fisher's exact test or the chi-square test. Continuous data was analyzed using a non-parametric test, i.e., the Mann-Whitney U test. We considered a p-value less than 0.05 as statistically significant. All statistical analysis was performed with GraphPad Prism 6^®^ (GraphPad Inc., La Jolla, USA).

## Results

**Figure 1:**
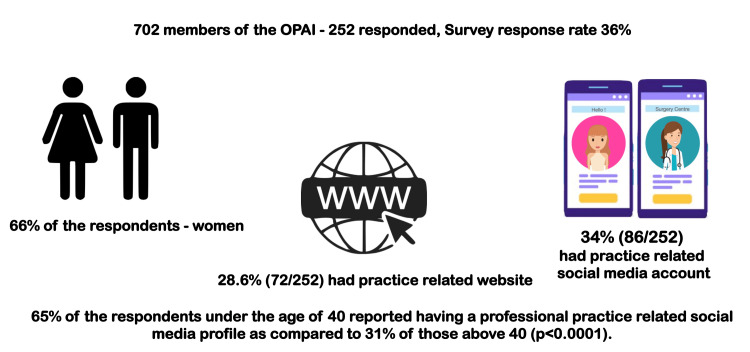
Infographic depicting the baseline demographic data of the respondents. WWW: World Wide Web Credit: Image created by the author

The survey was sent to 702 members of the OPAI, of which 252 responded, thus bringing the survey response rate to 36%. Of the 252 responses, 66% (166/252) were women (Figure 1).

Website ownership and social media presence

In all, 28.6% (72/252) of the respondents had their own professional practice-related website. Additionally, 34% (86/252) of the respondents had an active professional social media presence related to their practice. While comparing those who had a professional social media presence using age groups and genders of the respondents as variables, 65% of the respondents under the age of 40 reported having a professional practice-related social media profile as compared to 31% of those above 40. This difference was statistically significant (p<0.0001).

Of the 86 surgeons with social media accounts, 53 (61.6%) had separate personal and professional accounts, 12 (13.9%) respondents had only a professional practice-related account, and 21 (24.4%) members said they had only a personal account where they often posted work-related posts.

Perception of social media and medical practice

**Figure 2:**
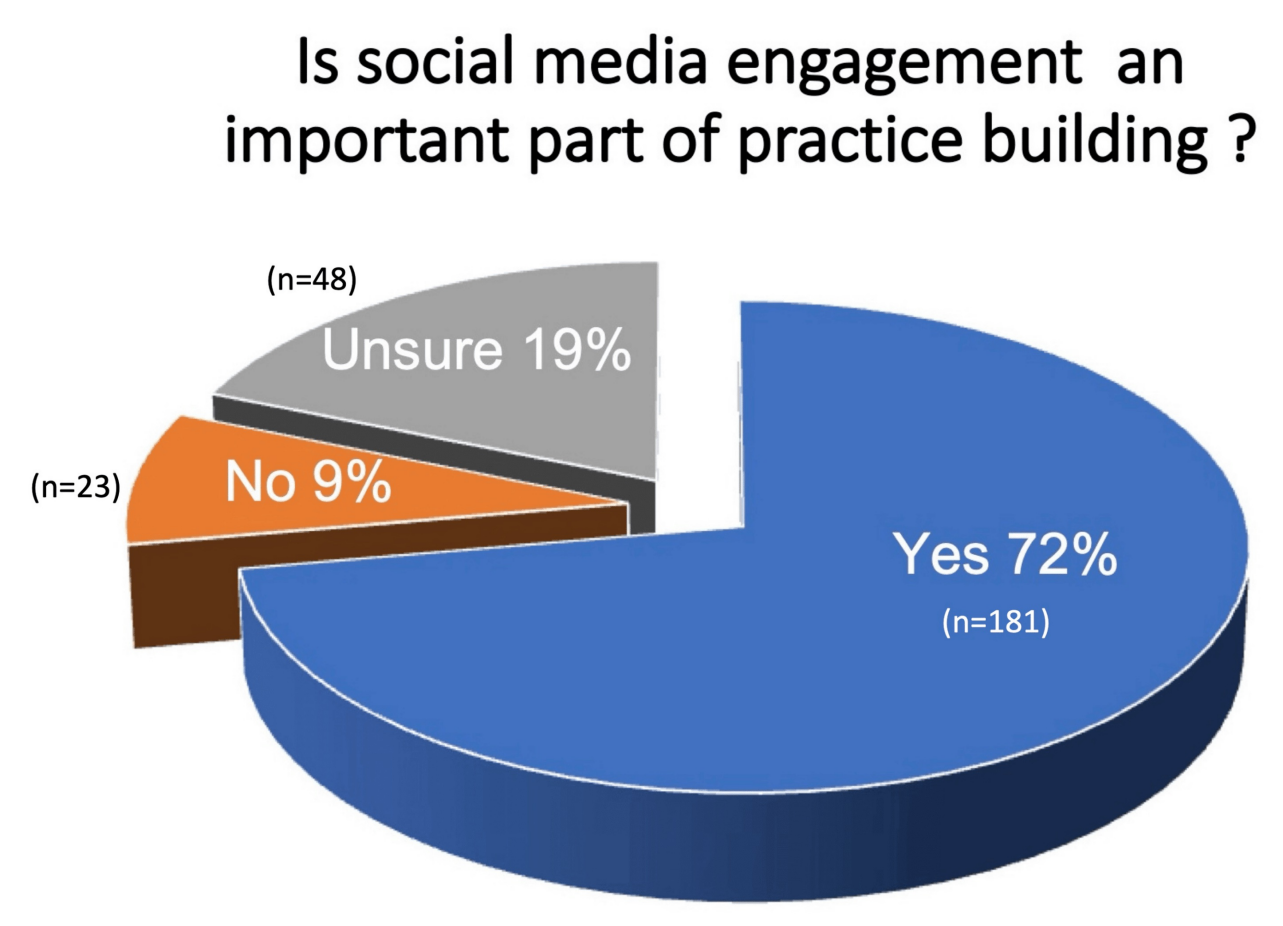
Responses of OPAI members to the question: Is social media engagement an important part of practice building? OPAI: Oculoplastics Association of India

When asked if they felt social media engagement was an important part of practice building, 72% of the respondents agreed, 9% disagreed, and 19% were unsure. The proportion of respondents under the age of 40 was significantly higher among those who felt that social media presence was an important part of practice building as compared to those who disagreed (51.6% vs. 31.4%; p<0.05) (Figure 2).

**Figure 3:**
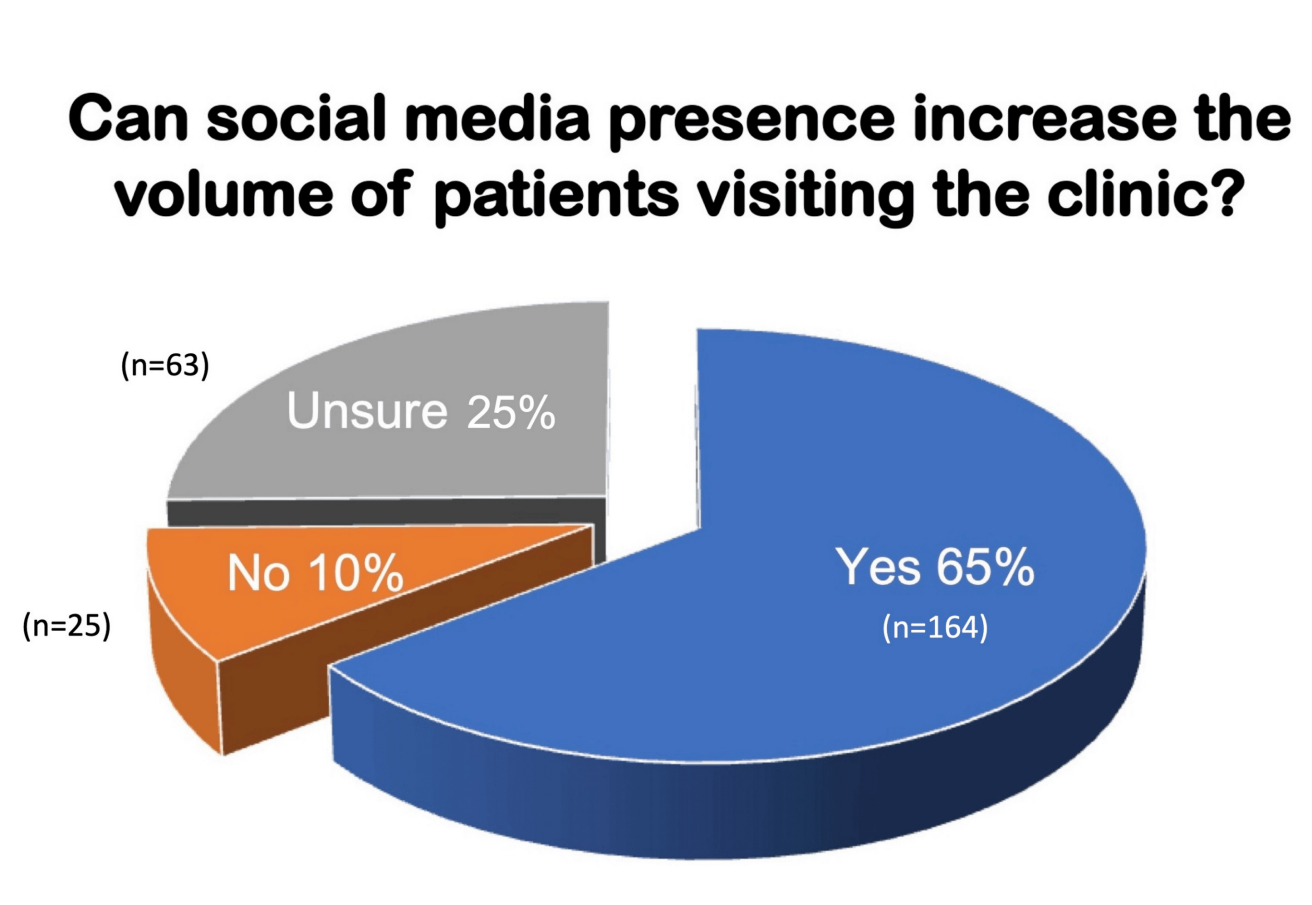
Visual representation of the respondents' responses to the question: Can social media presence increase the volume of patients visiting the clinic?

The respondents were also asked, "Can social media presence increase the volume of patients visiting their clinics?" Here, 63% (164/252) responded positively, 10% (25/252) responded negatively, and 25% (63/252) were not sure (Figure 3). Yet again, the proportion of respondents who were aged 40 or less was significantly higher among those who responded that social media engagement increases patient footfall (56.17% vs. 26.2%; p<0.05).

Social media platform preference

**Figure 4:**
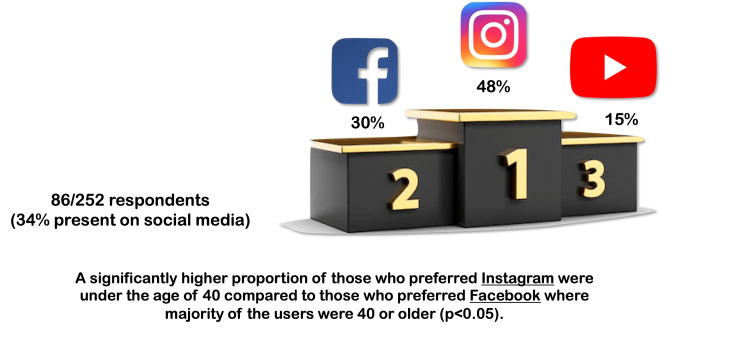
Infographic depicting the social media platform preference of the respondents. Credit: Image created by the author

The respondents were asked which social media platform they are most active on. The most popular platform was Instagram (41/86, 47.6%), followed by Facebook (26/86; 30.2%) and YouTube (13/86, 15.1%). While comparing the patterns of users who preferred Facebook versus Instagram, a significantly higher proportion of those who preferred Instagram were under the age of 40 compared to those who preferred Facebook, where the majority of the users were 40 or older (p<0.05) (Figure 4).

Results of social media marketing

**Figure 5:**
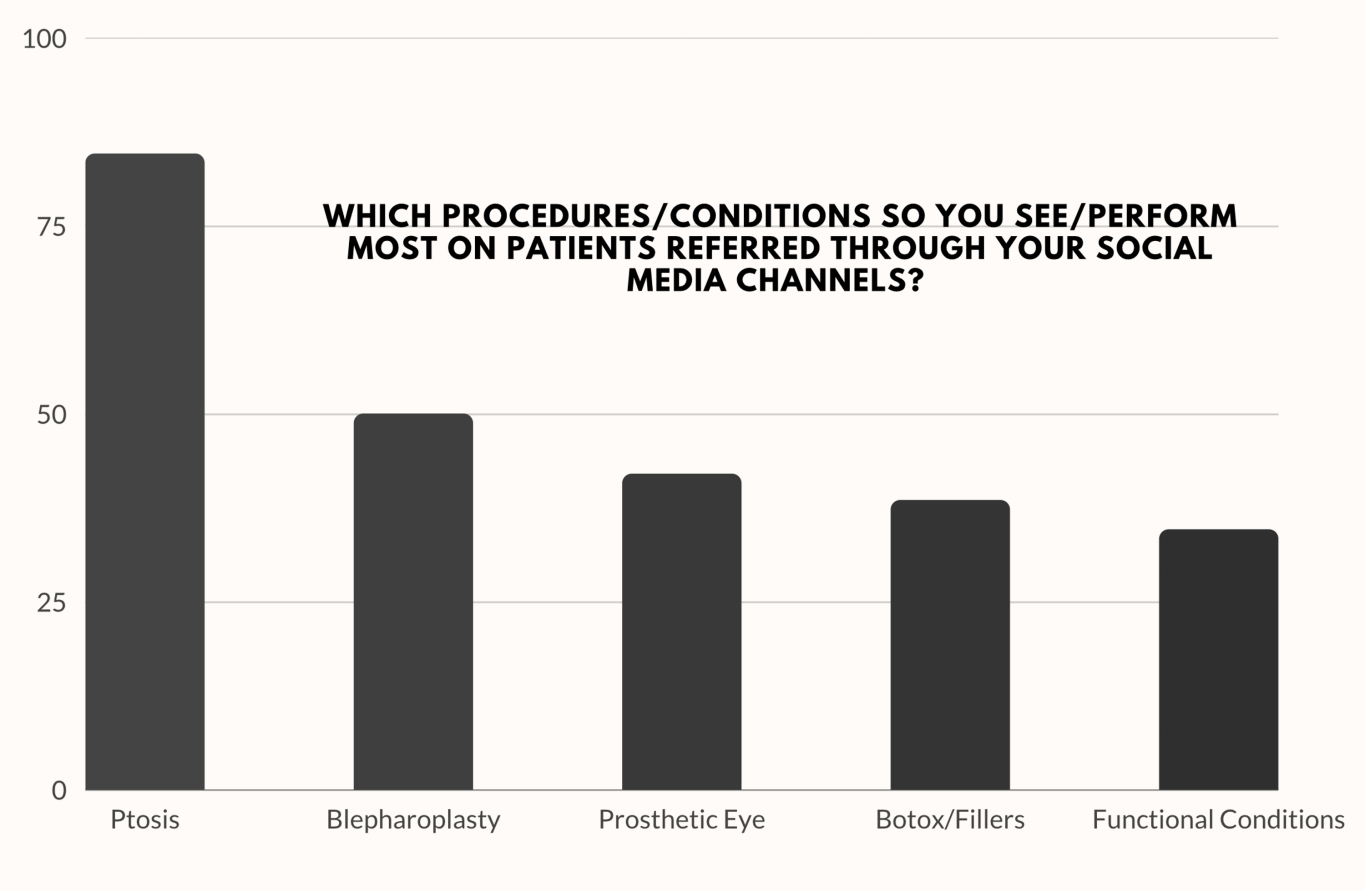
Most common conditions seen in clinic among patients self-referred through social media posts.

Additionally, 59.3% (51/86) of the surveyed surgeons with a social media presence reported that their social media presence resulted in patients scheduling appointments and clinic visits. In spite of Instagram being the most preferred social media platform for posts and engagement, when asked about which platform had helped the most in increasing visibility or improving practice, Facebook (37.3%) showed maximum results for the surgeons, followed by YouTube (27.5%) and Instagram (19.6%). The most common diagnosis/surgery seen amongst patients referred through social media was ptosis, with nearly 85% (44/51) of the respondents reporting increased patient visits for ptosis. This was followed by blepharoplasty (26/51; 50%), prosthetic eye fitting (22/51; 42%), non-invasive aesthetic procedures (20/51; 38.5%), and functional oculoplastic problems (18/51; 34.6%) (Figure 5).

The surgeons who did not have a social media presence were asked to enlist their reasons for not having a professional social media account. The most common response was that they "did not feel the need for a social media account related to their surgical practice," with 40% of the respondents indicating so. The next most commonly reported reason was the "pressure to post content regularly," which made them apprehensive about social media (35.3%). This was followed by "unfamiliarity with the platform" (22.4%), issues with patient consent (17.6%), "issues with archiving photographs" (14.1%), and "finding comparable before and after patient photographs" (11.8%).

## Discussion

Plastic surgery-related content on social media has an incredible reach and impact across the world, accounting for over 2 million posts, over 369 million likes, and 6.1 billion views seen across a 21-month period [3]. The trends in the use of social media platforms by different healthcare personnel have also changed over the past decade. Social media platforms earlier were used primarily for building professional networks, promoting journal publications, or sharing updates regarding conferences among peers and colleagues. Over a decade ago, in 2012, the most popular medical social media platform among medical professionals was Twitter. However, over the past decade, physicians and surgeons have realized the true potential for collaboration, education, and promotion on social media, where different topics can easily be searched through hashtags and threads. For example, plastic surgeons, dermatologists, and oculoplastic surgeons can use Facebook, Twitter, and Instagram to share de-identified patient information with before and after photographs to show the outcomes of surgeries and procedures [4]. The results of our survey show that oculoplastic surgeons in India may not have fully embraced and harnessed social media as a marketing tool. This is in stark contrast to oculoplastic, plastic, and cosmetic surgeons in the United States. One such survey showed that from 2011 to 2017, the percentage of American Society of Plastic Surgeons members with professional social media accounts increased from 28.2% to 61.9% [5]. With the number of medical professionals creating content as well as curating their own websites, there is no dearth of sources of information for patients. A previously published survey of healthcare workers reported that 90% of patients aged 18-24 years not only consumed the information on social media platforms but also believed health information from social media. Additionally, nearly 43% of healthcare workers reportedly encouraged their patients to read about disease processes on social media [6]. With doctors posting information regarding diseases, treatment options, surgical videos, recovery, and healing times as well as patient testimonials, social media has clearly become an important source of information that allows patients to better prepare and understand their visits to the clinic and subsequent procedures [7]. However, it is important to ensure that physicians adhere to the guidelines put in place by the local medical councils [8].

One recurring feature across all results in our survey was that younger oculoplastic surgeons in practice (those under the age of 40), apart from preferring Instagram over Facebook, were more active on social media and reportedly faced fewer problems technically with regard to posting content as well. The reasons for this are multifold: Instagram for the longest time was an exclusively mobile-only platform without a desktop interface. The content generation as well as the browsing experience was optimized for a mobile phone screen, which made it more popular amongst the younger generation, who are more technologically savvy when it comes to mobile phones.

In our survey, only 28% of the respondents had their own practice websites. Fan et al.’s survey showed that when asked which online method would be the most influential in selecting a surgeon, the practice website ranked the highest, followed by Google results, Facebook page, Instagram profile, and YouTube videos [5]. Additionally, Fan et al.’s survey highlighted that when the respondents (non-physicians) were asked if they would seek out a surgeon’s social media account before seeing the surgeon, 79% responded positively. One study that looked at the factors that influence decisions regarding facial cosmetic surgery among women in Saudi Arabia showed that advertisements posted by surgeons, exposure to social media, education, and a prior history of having undergone surgery are significant predictors [9].

While comparison with surveys in the West may not indicate the same patient preferences in India, one point that needs detailed discussion in the Indian context is the National Medical Council of India’s guidelines regarding social media use [8]. These guidelines on social media use by medical practitioners are considered a step toward promoting ethical behavior and protecting patient privacy. However, they introduce certain considerations that deserve further exploration. One aspect of these guidelines prohibits doctors from posting patients' photographs or scan images on social media, a rule primarily designed to safeguard patient privacy. Yet, these guidelines do not explicitly address the scenario where patients provide informed consent for sharing their images. This omission raises a critical ethical question: Should doctors be allowed to share patient images on social media if the patient consents? Sharing such images can have educational and awareness-raising benefits, as they can illustrate treatment outcomes or medical conditions at critical times, such as the mucormycosis outbreak in India during 2021-2022. To strike a balance between privacy and education, future revisions of these guidelines could incorporate provisions allowing for patient-consented image sharing, subject to stringent safeguards, such as written consent and complete anonymization. Additionally, these guidelines could benefit from more specific language to offer clearer guidance to medical practitioners. Overly vague or restrictive recommendations might discourage doctors from using social media for responsible educational purposes. The current guidelines may benefit from a more nuanced approach that permits patient-consented sharing of images and provides clearer guidance to doctors on responsible social media use for educational and awareness purposes. Balancing the imperatives of privacy and medical education in the digital age is a complex but essential task for medical regulatory bodies.

Clinical photography in general and, specifically, using smartphones in the clinic are acceptable for most patients [10]. Another aspect that needs to be studied in detail is the impact that social media has in shaping patient preferences as well as the effect of social media on mental health [11]. Social media, especially the use of artificial intelligence (AI) and filters, has the potential to create an unrealistic standard of beauty among normal populations. There is clearly a complex interplay between digital media exposure, altered self-perception, and subsequent increased inclination toward aesthetic procedures [12]. It is equally important to assess the potential ill effects of social media. The social media platform algorithms ensure a constant bombardment of similar images on one’s social media feed. This can slowly lead to body image dissatisfaction, imagined image defects, eventual body dysmorphic disorders, and the eventual pursuit of cosmetic surgery [13,14]. "Snapchat dysmorphia" and "selfie dysmorphia" are recognized terms for varying degrees of social media-induced dissatisfaction with appearance, and physicians must be cognizant of these trends [15].

This study has several strengths, including being the first survey to assess social media engagement among oculoplastic surgeons in India. It provides valuable insights into the evolving landscape of digital marketing in this specialty, particularly the significant age-based differences in social media usage.

However, the study also has limitations. The narrow focus on surgeons without patient perspectives may limit the generalizability of the findings. The gender skew (66% women respondents) and response rate (36%) may introduce bias, and the findings may not fully represent the entire population of oculoplastic surgeons in India. Additionally, the reliance on self-reported data may affect the accuracy of the results. The survey used in this study was developed based on a review of existing literature on social media usage in medical practice, particularly in surgical specialties. However, it was not subjected to formal validation or pilot testing prior to distribution. This limitation may affect the reliability and generalizability of the results. Additionally, while the survey's response rate is relatively low, it is consistent with typical response rates for online surveys in medical research. However, the potential for response bias must be acknowledged, as surgeons who are more engaged with social media may have been more likely to respond to the survey. This could result in an overestimation of social media engagement among oculoplastic surgeons in India. To mitigate this, future studies should consider validating the survey instrument and conducting pilot testing to ensure its reliability and validity.

## Conclusions

The study has some limitations including the limited response rate. Our study provides valuable insights into the landscape of social media engagement among oculoplastic surgeons in India. Younger OPAI members (<40 years) in India are more active on social media, with Instagram being their preferred platform, while those over 40 favor Facebook. OPAI members not active on social media face challenges such as pressure to post regularly and platform unfamiliarity. Ptosis and blepharoplasty are the primary conditions prompting patient outreach. While there is a growing recognition of the importance of social media in practice building, challenges related to unfamiliarity with the platform and the pressure to generate content persist. Nevertheless, those who actively engage in social media have reported some benefits in terms of increased clinic visits and improved visibility. Social media engagement positively correlates with increased patient numbers, particularly through Facebook, leading to more inquiries and visits. While a majority of the surgeons with a social media presence reported an increase in patient visits, it is important to note that this finding represents a correlation rather than causation. Other factors, such as an established reputation, geographic location, or word-of-mouth referrals, may also contribute to patient flow. Further studies are needed to establish a causal relationship between social media engagement and patient visits.

By understanding the various dynamics of social media usage, oculoplastic surgeons can potentially leverage social media more effectively to connect with patients and peers. Additionally, on an institutional scale, the OPAI could look at devising strategies to bridge the generational gap in social media engagement, ensuring that all members can harness the potential of digital platforms for their benefit.

## Appendices

Appendix 1: The questionnaire presented to the respondents, where all questions were mandatory.

1.      Is your oculoplastic practice based in India?

a.     Yes

b.     No

2.     Are you an OPAI Member?

a.     Yes

b.     No

3.     What is your age?

a.     <30

b.     31-40

c.     41-50

d.     >51

4.     What is your gender?

a.     M

b.     F

5.     What is the nature of your practice?

a.     Government/Municipal Hospital or Medical College

b.     Private Medical College

c.     Private Practice (Solo)

d.     Private Practice (Group / Chain / Multispecialty)

e.     Tertiary Care Institute

f.      Others

6.     Where is your practice location?

a.     Metropolitan City - Tier I - (Bangalore, Delhi, Chennai, Hyderabad, Mumbai, Pune, Kolkata, Ahmedabad)

b.     Tier II

c.     Tier III

d.     Rural

7.     Do you have your own professional website (Not including page on hospital/institute website)?

a.     Yes

b.     No

8.     Do you think social media presence is an important aspect of oculoplastic practice?

a.     Yes

b.     No

c.     Not Sure

9.     Do you think social media presence can help increase the volume of ones oculoplastics practice volume?

a.     Yes

b.     No

c.     Not Sure

10.  Do you have a social media presence related to your practice / professional account? (Twitter / Facebook / Instagram / YouTube / Quora / LinkedIn) 

a.     Yes

b.     No

11.  Do you have a separate professional page on any of the social media platforms?

a.     I have only a personal profile where I update professional posts

b.     I have only a professional profile

c.     I have a separate personal and professional profiles

12.  Which is the platforms are you present on where you post professional posts related to oculoplastic surgery? (you may choose more than one option)

a.     FaceBook

b.     Instagram

c.     Twitter/X

d.     YouTube

e.     Quora

f.      LinkedIn

g.     Others

13.  Which is the platform on which you are MOST ACTIVE on from a professional point of view? (choose only one)

a.     FaceBook

b.     Instagram

c.     Twitter/X

d.     YouTube

e.     Quora

f.      LinkedIn

g.     Others

14.  Do you manage your own social media platforms / website?

a.     Yes

b.     No, I have professional Help.

15.  What professional posts do you post on your social media platforms? (you may choose more than one)

a.     Case Outcomes - Before and After

b.     Complex cases for discussion

c.     Surgical videos

d.     Conference participation

e.     Patient testimonials

f.      Awards and achievements

g.     Personal posts

h.     Election campaigning

i.      Others

16.  Have your professional social media posts translated into patient visits to your clinic/hospital?

a.     Yes

b.     No

17.  Which platform has helped you the most in increasing visibility or improving practice?

a.     FaceBook

b.     Instagram

c.     Twitter/X

d.     YouTube

e.     Quora

f.      LinkedIn

g.     Others

18.  Which condition / surgery has social media increased referrals for you? (you may choose more than one)

a.     Ptosis

b.     Blepharoplasty

c.     Aesthetic procedures (Botox / Fillers)

d.     Prosthetic Eye / Shell

e.     Functional Conditions (DCR, Entropion, Ectropion)

f.      Orbital Decompression

g.     Ocular Oncology

h.     Others

19.  Any particular reason why you do not have a professional social media presence?

a.     Do not feel the need for a social media presence / Personal choice

b.     Archiving patient photos

c.     Finding before-after patient photos and creating posts

d.     Pressure to post content regularly

e.     Unfamiliarity with the platform

f.      Getting consent from patients to post photos

g.     Others

20.  Have you paid for promotional posts on social media platform? (advertisements / limited promotions / give-aways)

a.     Yes

b.     No

21.  What challenges do you face with your professional social media page? (You may choose more than one option)

a.     No Challenges

b.     Archiving patient photos

c.     Finding before-after patient photos and creating posts

d.     Pressure to post content regularly

e.     Unfamiliarity with the platform

f.      Others
